# Behavioral Disinhibition and Sexual Risk Behavior among Adolescents and Young Adults in Malawi

**DOI:** 10.1371/journal.pone.0073574

**Published:** 2013-09-09

**Authors:** Maureen Muchimba, Megan Burton, Sara Yeatman, Abdallah Chilungo, Brett C. Haberstick, Susan E. Young, Robin P. Corley, Matthew B. McQueen

**Affiliations:** 1 Institute for Behavioral Genetics, University of Colorado Boulder, Boulder, Colorado, United States of America; 2 Department of Integrative Physiology, University of Colorado Boulder, Boulder, Colorado, United States of America; 3 Department of Health and Behavioral Sciences, University of Colorado Denver, Denver, Colorado, United States of America; 4 Invest in Knowledge Initiative, Zomba, Malawi; Tulane University, United States of America

## Abstract

**Background:**

While behavioral factors such as early age of sexual debut, inconsistent use of condoms and multiple sexual partners have been studied in Africa, less is known about how characteristics such as impulsivity and externalizing behaviors relate to HIV-related sexual risk-taking in that region. The purpose of this study was to develop a culturally adapted behavioral disinhibition index in a sample of adolescents and young adults in Malawi. We then sought to examine the relationship between the index and sexual risk behavior as measured by multiple sexual partners and number of lifetime sexual partners.

**Methods:**

Cross-sectional data were collected from 2342 participants in rural Malawi aged 15 to 29 years. We constructed a disinhibitory behavior score (DBS) using questions assessing disinhibitory behaviors. Bivariate analyses were conducted to assess the relationships among the individual DBS component behaviors. We utilized multivariable logistic regression to determine the association of the DBS with multiple sexual partners, and negative binomial regression to model the relationship between the DBS and number of lifetime sexual partners.

**Findings:**

Nearly all the DBS component behaviors were significantly associated in the bivariate analyses. The DBS was associated with having multiple sexual partners (OR 1.97; 95% CI 1.57–2.48) in the multivariable logistic regression analysis. Further, negative binomial regression results demonstrated that the DBS was associated with an increased number of lifetime sexual partners (OR 1.11; 95% CI 1.07–1.16).

**Conclusions:**

HIV preventive programs in Africa should take into consideration disinhibitory behaviors that may be associated with sexual risk-taking. The DBS can be used as a simple tool to identify those who may be more likely to engage in these behaviors and provide useful information regarding which groups of individuals particularly need to be targeted for behavior change interventions.

## Introduction

Thirty-four million people are living with HIV/AIDS globally, with 2.5 million new infections in 2011 [1]. Although sub-Saharan Africa constitutes only 12% of the global population [2], it is home to 22.9 million adults and children living with HIV, representing 68% the global burden of HIV [1]. Within the region, Malawi bears one of the largest HIV-related disease burdens, with an HIV prevalence rate of 11%. It is estimated that 46,000 new infections occur annually in Malawi, 67% of which are among children and adults 15–49 years of age [1]. Among adolescents and young adults, HIV prevalence has been increasing for the past decade, owing in part to engaging in multiple sexual partnerships and concurrent relationships during these ages [3].Despite large investments of financial, political and intellectual efforts, new HIV infections continue at a rate that places sizable burdens on societies for decades to come. To stem the spread of HIV, it is imperative that public health initiatives provide comprehensive but targeted prevention messages [4]. To do so, behavioral factors relevant to HIV risk among younger age groups need to be identified and the relationships elucidated. While behavioral factors such as early age of sexual debut, inconsistent use of condoms and multiple sexual partners have been implicated in the spread of HIV globally, so have impulsivity, substance misuse and externalizing behaviors [5]–[7]. However, aside from research from industrialized nations, there is little understanding of how the latter behaviors relate to HIV risk in populations from developing countries, more specifically, in Malawian adolescents and young adults.

Behavioral disinhibition is a construct that indexes an individual’s inability to inhibit socially undesirable or restricted behaviors. Features of this index include impulsivity and sensation-seeking, externalizing psychopathology such as the violation of rules and the rights of others, and licit and illicit drug use [8]–[11]. Individuals who score at the high end of the behavioral disinhibition spectrum are hyposensitive to the negative consequences of socially undesirable behaviors, even in the face of adverse educational, economic, interpersonal and legal consequences. Although specific traits related to behavioral disinhibition, such as sensation-seeking and impulsivity, have been linked to an increased frequency of HIV-related sexual risk taking behaviors, including unsafe sexual practices such as multiple partners [12], [13], research also demonstrates associations between clustered disinhibitory behaviors and adverse health outcomes. In one study, engaging in behavior that increased the risk of HIV and other sexually transmitted infections was significantly related to antisocial behavior, which was measured by aggressive behavior and delinquent acts such as stealing and vandalism [14]. In a prospective study of men and women, problem behavior at age 15, which included items measuring conduct problems, aggressive delinquency and substance abuse, was an antecedent of intimate partner violence perpetration at age 21 [15].

Although behavioral disinhibition has been studied in developed nations, less is known about this construct in non-Western populations. It is unclear how it manifests in populations from developing country settings and its relationship to sexual risk behavior in these populations remains largely unknown. Further, it is not clear whether findings on the association between behavioral disinhibition and sexual risk behavior from Western societies are generalizable to populations from developing regions. To our knowledge, there are no studies on behavioral disinhibition in sub-Saharan Africa, particularly in areas that are burdened with a high prevalence of HIV, such as Malawi. The purpose of this study was to develop a culturally adapted behavioral disinhibition index in a sample of adolescents and young adults in Malawi. We then sought to examine the relationship between the index and sexual risk behavior, measured by multiple sexual partners and number of lifetime sexual partners.

## Materials and Methods

### Ethics Statement

All participants provided written informed consent (18 years and older) or written informed assent (younger than 18 years). Written consent was also obtained from the parents or guardians of those younger than 18 years. All study protocols were approved by the Pennsylvania State University Office for Research Protections, the Malawi National Health Sciences Research Committee and the University of Colorado Boulder Institutional Review Board.

### Sample

We drew our sample of from the Tsogolo la Thanzi (TLT) project [16], an on-going longitudinal study of young women and men, and the male partners of the female respondents in Balaka, a rural district in Malawi. Briefly, approximately 1,500 female and 600 male respondents were randomly selected from a sampling frame of residents in census enumeration areas within a seven-kilometer radius of Balaka. At the baseline interview and at each subsequent wave, women were asked to report on their on-going relationships and any new partnerships, and new male partners of the women were invited to participate in the study. Participants were interviewed every four months. This study primarily utilized wave 4 data, collected between June and August 2010, and was restricted to participants aged 15 to 29 (n = 2342).

### Materials

We developed a short questionnaire–the TLT-Risk Behavior Questionnaire (TLT-RBQ)–for use in the Malawi sample to assess disinhibitory and substance use behaviors. The TLT-RBQ was administered in conjunction with the primary survey questionnaires of the TLT project during the 4th wave of the overall project. The items in the TLT-RBQ are comparable to questions from the Composite International Diagnostic Interview-Substance Abuse Module [17], the South Oaks Gambling Screen [18] and the Monitoring the Future questionnaire [19].

### Measures

#### Behavioral disinhibition

We constructed a scored variable, the disinhibitory behavior score (DBS), which was the predictor variable of interest. Behavioral disinhibition was assessed using six items: having first drunk alcohol prior to age 16, lifetime licit and illicit substance use, fighting, theft, vandalism and gambling. Each item was dichotomized according to whether or not a participant ever engaged in that behavior and then summed to create an overall measure of behavioral disinhibition, with scores ranging from 0 to 6. The DBS was compared to an existing disinhibition index created from an all-white, American sample from the Center for Antisocial Drug Dependence (CADD), a large longitudinal study of twins, adopted individuals, and their families in Colorado [20]–[22]. The intraclass correlation coefficient between the DBS and the index from the CADD sample was 0.54, indicating a moderate correlation.

#### Sexual risk behavior

Sexual risk behavior was measured by multiple sexual partners and number of lifetime sexual partners. High numbers of sexual partners represents a key risk factor for HIV transmission [23], [24], as greater numbers increase the probability that any one random act of intercourse will result in infection [25]. Multiple sexual partners was assessed with a single question asking whether the respondent had two or more sexual partners in the previous four months. A positive response was coded as 1, while those reporting fewer than two partners were coded as 0. Participants were also asked how many sexual partners they had had in their lifetime.

### Statistical Analysis

We first ran bivariate analyses to examine the associations among the individual disinhibitory behaviors that constituted the DBS. The association between the DBS and multiple sexual partners was evaluated using multivariable logistic regression and the model controlled for age, gender, marital status and education level. We treated the number of lifetime sexual partners as a count variable. However, given the non-independence of the counts (i.e. a participant may report more than one lifetime sexual partner), we further approached it as an overdispersed count variable. To accommodate the analysis of this type of variable, we adopted a negative binomial regression to model the relationship between the DBS and lifetime sexual partners, controlling for age, gender, marital status and education level. We also considered a zero-inflated negative binomial model and conducted a Vuong test to compare the negative binomial to a zero-inflated negative binomial model. The Vuong test indicated that the negative binomial was a better fit to the data. Primary statistical analyses were conducted using SAS software (version 9.3; SAS Institute, Cary, NC). To test between the negative binomial and zero-inflated negative binomial we used the “MASS” package in R [26].

## Results

### Descriptive Statistics

The characteristics of the study participants are displayed in Table 1. The majority of the sample was female (57.5%) and was aged 18 to 23 years, with a mean age of 21.2 (SD = 3.6). Slightly over half of the sample (53.5%) was currently not married. Most of the respondents (58.5%) attained primary school education, while less than 2.0% had no formal education, and even fewer advanced beyond secondary school. The most prevalent disinhibitory behaviors were fighting and gambling, with each behavior being reported by approximately 16% of the sample. Less than 10% of respondents had a young age (<16 years) at first, drink while only 3.2% reported ever using drugs.

**Table 1:** Sociodemographic and risky behavior characteristics of the study sample.

Characteristic	n (%)		
	Female	Male	Total
Age			
15–17	328 (24.35)	138 (13.87)	466 (19.90)
18–20	338 (25.09)	239 (24.02)	577 (24.64)
21–23	350 (25.98)	223 (22.41)	573 (24.47)
24+	331 (24.57)	395 (39.70)	726 (31.00)
Marital status			
Currently married	661 (49.07)	428 (43.02)	1089 (46.50)
Currently not married	686 (50.93)	567 (56.98)	1253 (53.50)
Education level			
No formal education	30 (2.26)	9 (0.91)	39 (1.68)
Primary school	840 (63.21)	516 (52.23)	1356 (58.52)
Secondary school	456 (34.31)	457 (46.26)	913 (39.40)
Tertiary school	3 (0.23)	6 (0.61)	9 (0.39)
Young age at first drink			
No	1316 (97.84)	812 (82.10)	2128 (91.17)
Yes	29 (2.16)	177 (17.90)	206 (8.83)
Ever used drugs			
No	1336 (99.48)	925 (93.25)	2261 (96.83)
Yes	7 (0.52)	67 (6.75)	74 (3.17)
Ever gambled			
No	1201 (89.16)	776 (77.99)	1977 (84.42)
Yes	146 (10.84)	219 (22.01)	365 (15.58)
Ever been involved in theft			
No	1200 (89.09)	927 (93.17)	2127 (90.82)
Yes	147 (10.91)	68 (6.83)	215 (9.18)
Ever vandalized			
No	1313 (97.48)	973 (97.79)	2286 (97.61)
Yes	34 (2.52)	22 (2.21)	56 (2.39)
Ever been in a fight			
No	1173 (87.15)	788 (79.20)	1961 (83.77)
Yes	173 (12.85)	207 (20.80)	380 (16.23)
Multiple sexual partners			
No	1338 (99.33)	958 (96.28)	2296 (98.04)
Yes	9 (0.67)	37 (3.72)	46 (1.96)

### Associations among Disinhibitory Behaviors

Table 2 presents the associations, via odds ratios (OR) and 95% confidence intervals (CI), among the different components of the DBS. Nearly all the specific behaviors were positively associated with each of the other behaviors in the bivariate analyses. For example, a participant is seven times more likely to engage in fighting if they have also reported ever using drugs (OR 7.06; 95% CI 4.40–11.32). The strongest association was between vandalism and theft (OR 16.5; 95% CI 9.5–28.7).

**Table 2:** Odds ratios and 95% confidence intervals of unadjusted associations among components of the DBS.

	Age at first drink (<16)	Drug use	Fighting	Gambling	Theft	Vandalism
Age at first drink (<16)						
Drug use	9.17 (5.65–14.91)					
Fighting	2.17 (1.57–3.01)	7.06 (4.40–11.32)				
Gambling	1.88 (1.34–2.64)	4.17 (2.59–6.71)	2.26 (1.74–2.94)			
Theft	1.47 (0.95–2.27)	3.13 (1.79–5.49)	3.56 (2.63–4.81)	3.20 (2.35–4.35)		
Vandalism	0.57 (0.21–1.23)	1.80 (0.56–5.90)	4.05 (2.36–6.91)	3.96 (2.26–6.82)	16.52 (9.50–28.73)	

### Disinhibitory Behavior Score

**Figure 1:**
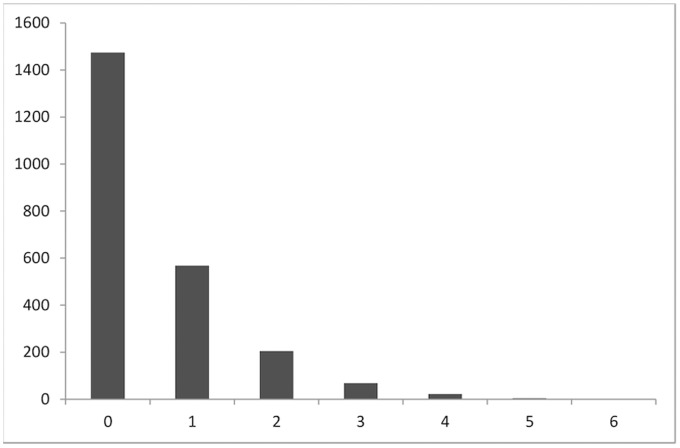
Frequency distribution of disinhibitory behavior scores.

The distribution of the DBS is presented in Figure 1. The modal score was 0 or no disinhibitory behavior, reported by 62.0% of the sample. Among those who engaged in any disinhibitory behavior, 24.8% reported only one, while only 1.2% had engaged in four or more behaviors. More males than females reported ever engaging in any of the behaviors.

### Sexual Risk Behavior

#### Multiple sexual partners

Approximately 2.0% of the respondents reported having multiple sexual partners over the relatively short (∼4 months) reference period. Nevertheless, as shown in Table 3, after controlling for age, gender, marital status and education level, each one-unit increase on the DBS was associated with a nearly two-fold increase in the likelihood of having multiple sexual partners in the previous four months (OR 1.97; 95% CI 1.57–2.48). Among respondents who scored 0 on the DBS, only 12 (0.5%) reported having multiple sexual partners in the previous four months. The only other variable significantly associated with multiple sexual partners was gender, with females being less likely to report multiple sexual partners (OR 0.21; 95% CI 0.09–0.47).

**Table 3:** Logistic regression analyses of the relationship between the DBS and multiple sexual partners.

Characteristic	OR (95% CI)	
	Crude	Adjusted
DBS	2.19 (1.77–2.72)	1.97 (1.57–2.48)
Age	1.00 (0.92–1.08)	0.95 (0.85–1.07)
Gender		
Male	1.0	1.0
Female	0.17 (0.08–0.36)	0.21 (0.09–0.47)
Married		
Yes	1.0	1.0
No	1.65 (0.89–3.03)	0.98 (0.42–2.26)
Education level		
Secondary or higher	1.0	1.0
Primary or less	0.52 (0.29–0.95)	0.68 (0.36–1.29)

#### Lifetime sexual partners

**Figure 2:**
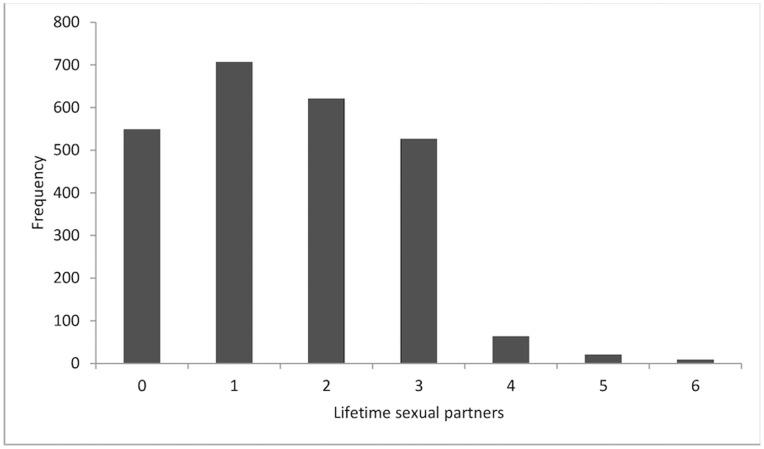
Frequency distribution of the number of lifetime sexual partners.

The modal number of lifetime sexual partners was one (Figure 2). Twenty-two percent reported having no sexual partner in their lifetime, while 24.9% had more than two partners. As noted in the statistical analysis section, we determined that a negative binomial regression was a better fit than a zero-inflated negative binomial regression. Results from the negative binomial regression modeling lifetime sexual partners as an overdispered count variable are presented in Table 4. The number of lifetime sexual partners increased by approximately 11% with every one-unit increase in the DBS (OR 1.11; 95% CI 1.07–1.16). Similar to the results for multiple sexual partners, female gender was negatively associated with the number of lifetime sexual partners (OR 0.79; 95% CI 0.73–0.84). Those who were currently unmarried were also less likely to report a high number of lifetime partners (OR 0.73; 95% CI 0.67–0.79), while the likelihood of having a higher number of lifetime partners increased with age (OR 1.09; 95% CI 1.08–1.10).

**Table 4:** Negative binomial regression analyses of the relationship between the DBS and the number of lifetime sexual partners.

Characteristic	OR (95% CI)	
	Crude	Adjusted
DBS	1.15 (1.11–1.19)	1.12 (1.08–1.16)
Age	1.12 (1.11–1.14)	1.09 (1.08–1.10)
Gender		
Male	1.00	1.00
Female	0.67 (0.62–0.71)	0.79 (0.73–0.84)
Married		
Yes	1.00	1.00
No	0.53 (0.49–0.57)	0.73 (0.67–0.79)
Education level		
Secondary or higher	1.00	1.00
Primary or less	1.01 (0.95–1.08)	1.04 (0.97–1.12)

## Discussion

This study sought to develop a culturally adapted behavioral disinhibition index in a sample of adolescents and young adults in Malawi, and to examine the relationship between the index and sexual risk behavior as measured by multiple sexual partners and number of lifetime sexual partners. It is one of the few studies to assess a measure of behavioral disinhibition in an African population.

Disinhibitory behavior was not highly prevalent in this study sample. Although results showed rates of approximately 16% for fighting and gambling, the literature reports higher rates of these behaviors in other parts of the world. For example, a study among young people in the southern region of the United States showed that 18.7% and 8.6% of males and females, respectively, were involved in a physical fight more than once during the previous month [27]. In a nationally representative sample of Swedish youth aged 16–24 years, the prevalence of past-year gambling was 43.1% among males and 39.4% among females [28]. It is important to note, however, that the sample in the present study was from a rural area in Malawi, which could account for the lower levels of disinhibitory behaviors. It is possible that the prevalence of these behaviors is higher in urban African populations. Despite the lower prevalence of these behaviors, the significant associations observed among the disinhibitory behaviors in this rural Malawian population provide evidence that these behaviors tend to express in a cluster-correlated manner in this sub-Saharan study population as has been previously demonstrated in Western populations [9], [29]. The fact that similar findings have been shown in different populations emphasizes the claim that descriptive differences do not necessarily entail differences in relationships among variables at the underlying, causal or explanatory level [30].

Consistent with studies that have found associations between sexual risk behavior and personality traits related to behavioral disinhibition, such as impulsivity and sensation-seeking [13], the DBS was associated with an increased likelihood of having multiple sexual partners and having a higher number of lifetime sexual partners. This suggests that individuals who do not inhibit certain behaviors because of an inability for restraint, thrill-seeking or a lack of concern about the consequences are less likely to be inhibited in their sexual behavior. The findings that these associations were observed in a setting where disinhibitory behaviors were very low highlight the usefulness of methods such as the DBS in characterizing individuals who are likely to be more susceptible to HIV-related sexual behavior. These observations are especially salient for sub-Saharan countries like Malawi, where multiple and concurrent sexual partners are believed to be a major driving force in the HIV epidemic. Findings such as ours may be integrated into already effective intervention and prevention public health initiatives in sub-Saharan Africa. While these programs are unlikely to modify personality, they may increase their effectiveness by looking beyond HIV-related behaviors to factors that underlie these behaviors as they design their preventive strategies. Rather than adopting a one-size-fits-all approach, health promotion programs can make use of information related to individuals’ predisposition to disinhibitory behaviors so that messages can be tailored to target different personality types.

This study has several limitations. First, our sample was drawn from a small rural district in Malawi and does not represent Malawi as a nation. Inferences from these data can only be made for the sample assessed and the limited populations it may represent. The second limitation stems from the non-random nature of the sample, which included male partners of the female participants. Caution should be taken when interpreting the results due to the potential for biases such as participation bias. Third, the data rely on retrospective self-reported data. Therefore, there was a possibility of participants having difficulty recalling important information or providing socially desirable responses to the sensitive questions, leading to respondent bias. Fourth, the measure of multiple sexual partners, as two or more partners in the previous four months may not be the most appropriate measure of HIV risk. Fifth, cultural differences in questions assessing the type, availability and use patterns of licit and illicit drugs, as well as considerations of what constitutes conduct-related problems, may have limited our ability to measure the same qualities between the DBS and a behavioral disinhibition scale derived in North America. Along these lines, we would expect that a broader inclusion of culturally sensitive questions that facilitate the ascertainment of locally relevant problem drug use and conduct behaviors would improve the ascertainment of individuals at higher risk for HIV infection and/or transmission. Lastly, our data were cross-sectional; therefore, a temporal relationship cannot be established. In spite of these limitations, the findings of our study demonstrate that behavioral disinhibition measures from Western societies can be culturally adapted to developing country populations. Further, they strengthen the claim for the generality of the relationship between behavioral disinhibition and other risk-taking behaviors beyond industrialized populations.

## Conclusions

A culturally adapted behavioral disinhibition index was associated with sexual risk behavior among adolescents and young adults in rural Malawi. Our findings have important public health implications for HIV preventive programs in Africa by providing insights into the key role that individual characteristics play in sexual risk-taking behaviors, such as multiple sexual partners. The DBS can be used as a simple screening method to identify those who may be more likely to engage in these behaviors, and can provide useful information regarding which groups of individuals particularly need to be targeted for behavior change interventions. Future studies can build on this research by including a more extensive range of culturally sensitive questions in order to better assess locally relevant disinhibitory behaviors and by examining behavioral disinhibition and its relationship to sexual risk behavior in urban African samples, where HIV rates may be higher.
